# ECG-Based Indices to Characterize Persistent Atrial Fibrillation Before and During Stepwise Catheter Ablation

**DOI:** 10.3389/fphys.2021.654053

**Published:** 2021-03-30

**Authors:** Anna McCann, Jean-Marc Vesin, Etienne Pruvot, Laurent Roten, Christian Sticherling, Adrian Luca

**Affiliations:** ^1^Applied Signal Processing Group, Swiss Federal Institute of Technology, Lausanne, Switzerland; ^2^Service of Cardiology, Lausanne University Hospital and University of Lausanne, Lausanne, Switzerland; ^3^Department of Cardiology, Inselspital, Bern University Hospital, University of Bern, Bern, Switzerland; ^4^Department of Cardiology, University Hospital Basel, Basel, Switzerland

**Keywords:** atrial fibrillation, electrocardiographic markers, outcome stratification, catheter ablation, temporal analysis

## Abstract

**Background:** Consistently successful patient outcomes following catheter ablation (CA) for treatment of persistent atrial fibrillation (pers-AF) remain elusive. We propose an electrocardiogram (ECG) analysis designed to (1) refine selection of patients most likely to benefit from ablation, and (2) examine the temporal evolution of AF organization indices that could act as clinical indicators of ongoing ablation effectiveness and completeness.

**Method:** Twelve-lead ECG was continuously recorded in 40 patients (61 ± 8 years) during stepwise CA (step-CA) procedures for treatment of pers-AF (sustained duration 19 ± 11 months). Following standard pre-processing, ECG signals were divided into 10-s epochs and labeled according to their temporal placement: pre-PVI (baseline), dur-PVI (during pulmonary vein isolation), and post-PVI (during complex-fractionated atrial electrograms and linear ablation). Instantaneous frequency (IF), adaptive organization index (AOI), sample entropy (SampEn) and f-wave amplitude (FWA) measures were calculated and analyzed during each of the three temporal steps. Temporal evolution of these measures was assessed using a statistical test for mean value transitions, as an indicator of changes in AF organization. Results were then compared between: (i) patients grouped according to step-CA outcome; (ii) patients grouped according to type of arrhythmia recurrence following the procedure, if applicable; (iii) within the same patient group during the three different temporal steps.

**Results:** Stepwise CA patient outcomes were as follows: (1) left-atrium (LA) terminated, not recurring (LTN, *n* = 8), (2) LA terminated, recurring (LTR, *n* = 20), and (3) not LA terminated, all recurring at follow-up (NLT, *n* = 12). Among the LTR and NLT patients, recurrence occurred as AF in seven patients and atrial tachycardia or atrial flutter (AT/AFL) in the remaining 25 patients. The ECG measures indicated the lowest level of organization in the NLT group for all ablation steps. The highest organization was observed in the LTN group, while the LTR group displayed an intermediate level of organization. Regarding time evolution of ECG measures in dur-PVI and post-PVI recordings, stepwise ablation led to increases in AF organization in most patients, with no significant differences between the LTN, LTR, and NLT groups. The median decrease in IF and increase in AOI were significantly greater in AT/AFL recurring patients than in AF recurring patients; however, changes in the SampEn and FWA parameters were not significantly different between types of recurrence.

**Conclusion:** Noninvasive ECG measures, though unable to predict arrhythmia recurrence following ablation, show the lowest levels of AF organization in patients that do not respond well to step-CA. Increasing AF organization in post-PVI may be associated with organized arrhythmia recurrence after a single ablation procedure.

## 1. Introduction

Despite the widespread use of catheter ablation (CA) therapy in treating atrial fibrillation (AF), its long-term efficacy and benefit in improving clinical outcomes are particularly disputed when used to treat patients suffering from persistent AF (pers-AF) (Nademanee et al., 2004; Brooks et al., 2010). The recurrence of AF following CA treatment remains unexplained, in part due to an incomplete understanding of the underlying electrophysiological mechanisms that trigger and sustain arrhythmias (Schotten et al., 2011).

Several studies have used surface electrocardiogram (ECG) and intracardiac electrogram (EGM) measures to characterize AF complexity and predict lack of AF recurrence following CA, with limited success (Vikman et al., 2003; Matsuo et al., 2009; Bonizzi et al., 2010; Guillem et al., 2013). While higher dominant frequency (DF) and lower organization index (OI) values have been associated with recurrence of AF, these time-invariant measures reflect only the average tendencies of the signal. Additionally, little work has investigated the temporal evolution of ECG parameters throughout CA, focusing rather on their values prior to ablation, or at a limited number of time segments in each CA step. To allow for a thorough investigation of the temporal evolution, this study used signals recorded throughout the entire procedure, and analyzed them using time-variant measures that account for the highly non-stationary nature of ECG signals recorded in AF. We first report the average tendencies of these time-variant parameters at each CA ablation step, and then analyze in further detail their evolution within each step.

## 2. Materials and Methods

### 2.1. Study Population

Forty patients suffering from pers-AF (38 M/ 2 F, 61 ± 8 years) who were referred for a first ablation after displaying resistance to pharmacological and electrical cardioversion interventions were included prospectively in the study. The patients were suffering from AF for 6 ± 4 years, sustained for 19 ± 11 months before ablation. All patients discontinued antiarrhythmic drugs for at least five half-lives prior to the CA, except for amiodarone and beta-blockers. Oral anticoagulation was prescribed for at least one month prior to the procedure. Stepwise CA (step-CA) was performed under general anesthesia (GA) by a single operator (EP) at the Lausanne University Hospital as described in Buttu et al. (2013).

The protocol prescribed a stepwise approach, beginning with pulmonary vein isolation (PVI), followed by left atrial (LA) complex fractionated atrial electrogram (CFAE) ablation, and ending with LA linear ablation (roof and mitral isthmus). Table 1 provides an overview of patient clinical characteristics. Following the first procedure, patients who experienced a recurrence of atrial arrhythmia could undergo repeat procedures. Twelve-lead surface ECGs were continuously recorded for off-line analysis at a sampling frequency of 2 kHz (Axiom Sensis XP, Siemens) at pre-PVI (i.e., before ablation), as well as throughout the step-CA procedure. ECG chest lead *V*_6_ was placed on the backs of patients (*V*_6*b*_), to better capture LA activity (Petrutiu et al., 2009). The study protocol was approved by the Lausanne University Hospital Human Research Ethics Committee, and all patients provided written informed consent.

**Table 1:** Patient clinical characteristics. Values are expressed as median with [25th; 75th] percentiles or as patient counts (*n*) and percentages of total patient count.

	**LTN**	**LTR**	**NLT**	***p*-value^a^**	***p*-value^b^**	***p*-value^c^**
	***n* = 8**	***n* = 20**	***n* = 12**			
Age (yrs)	60 [58; 63]	63 [53; 67]	63 [58;64]	0.65	0.56	0.98
Sex (male/female)	8/0	18/2	12/0	0.35	–	0.26
AF duration (yrs)	9 [6; 11]	5 [3; 6]	5 [2; 7]	0.07	**0.03**	0.74
Duration of sustained AF (mo)	20 [10; 24]	13 [12; 24]	22 [14; 39]	0.6	0.28	0.05
Body mass index (kg/m2)	29 [27; 30]	30 [25; 34]	30 [24; 31]	0.72	0.94	0.63
High blood pressure	6 (75)	13 (65)	8 (67)	0.68	0.69	0.72
Valvular disease	2 (25)	3 (15)	1 (8)	0.53	0.31	0.58
Diabetes	1 (13)	3 (15)	2 (17)	0.86	0.8	0.9
Tobacco	2 (25)	4 (20)	2 (17)	0.77	0.65	0.81
Hypercholesterolemia	3 (38)	9 (45)	6 (50)	0.72	0.58	0.78
Coronary artery disease	0 (0)	0 (0)	0 (0)	–	–	–
Sleep apnea syndrome	4 (50)	13 (65)	6 (50)	0.46	>0.99	0.4
Chronic kidney disease	0 (0)	1 (5)	0 (0)	0.52	–	0.43
CHA2DS2-Vasc score	1 [1; 2]	2 [1; 2]	1 [0; 1]	0.47	0.41	0.12
Dilated cardiomyopathy	2 (25)	9 (45)	3 (25)	0.33	>0.99	0.26
Hypertrophic cardiomyopathy	1 (13)	2 (10)	0 (0)	0.85	0.21	0.26
Left ventricular fraction ejection (%)	55 [44; 55]	50 [35; 56]	55 [50; 60]	0.47	0.5	0.18
Left atrial volume (ml)	167 [139; 204]	162 [140; 190]	174 [160; 193]	0.9	0.64	0.41
Beta-blockers	6 (75)	15 (75)	7 (58)	>0.99	0.44	0.33
Calcium channel blockers	2 (25)	4 (16)	2 (17)	0.77	0.65	0.82
Amiodarone	2 (25)	4 (16)	1 (8)	0.77	0.31	0.38
Other antiarrhythmics	0 (0)	2 (10)	3 (25)	0.35	0.13	0.26
Enzyme conversion inhibitor	2 (25)	3 (15)	3 (25)	0.53	>0.99	0.48
Angiotensin receptor inhibitor	0 (0)	4 (20)	1 (8)	0.17	0.4	0.38
No. of antiarrhythmics drugs	2 [2; 2]	3 [2; 3]	2 [2; 3]	0.43	0.27	**0.03**
Statins	0 (0)	2 (10)	3 (25)	0.35	0.13	0.26
**Cumulative ablation time (min)**						
Total	40 [29; 54]	55 [50; 60]	76 [61; 81]	**0.04**	**<0.01**	**<0.01**
Pulmonary vein isolation	22 [16; 25]	25 [20; 28]	19 [16; 23]	0.39	0.76	0.11
CFAEs and linear ablation	19 [5; 30]	26 [20; 40]	55 [48; 59]	0.09	**<0.01**	**<0.01**
**Recurrence type at follow-up**						
Persistent AF		1	3			
Paroxysmal AF		3	0			
AT/AFL		16	9			

*AF, atrial fibrillation; AT, atrial tachycardia; AFL, atrial flutter; CFAEs, complex fractionated atrial electrograms; LTN, left-terminated, not recurring at follow-up; LTR, left-terminated, recurring at follow-up; NLT, not left-terminated*.

a *LTN vs. LTR*.

b *LTN vs. NLT*.

c *LTR vs. NLT*.

### 2.2. Procedure and Clinical Outcomes

Termination of AF into either sinus rhythm (SR) or atrial tachycardia (AT) at any step of the ablation constituted the procedural endpoint. When this procedural endpoint was not reached, electrical cardioversion was performed. Clinical follow-up, including 48-h Holter recordings, was performed at scheduled visits 3, 6, 12, and 18 months, then 2, 3, and 4 years after the initial ablation procedure to monitor arrhythmia recurrence, defined as AT, atrial flutter (AFL), or AF lasting more than 30 s (Calkins et al., 2018) observed during the follow-up period.

Based on the observed procedural and clinical outcomes, the study population was divided into the following three groups: (1) left terminated, non-recurring (LTN, *n* = 8), patients in whom AF was terminated into SR or AT at any stage of step-CA, and who remained arrhythmia free throughout the clinical follow-up period; (2) left terminated, recurring (LTR, *n* = 20), patients in whom AF was terminated into SR or AT, and who experienced a recurrence after a single step-CA procedure throughout follow-up; (3) not left-terminated (NLT, *n* = 12), patients in whom the step-CA procedure failed to terminate pers-AF. In the case that an arrhythmia recurrence occurred (LTR and NLT groups), the recurrence type was labeled as AT/AFL or AF.

### 2.3. ECG Pre-processing and Atrial Activity Extraction

Signal processing was performed in MATLAB™. ECG signals were visually inspected before processing, and signals containing excessive noise or artifacts were removed from the analysis. Prior to atrial activity extraction, a fifth order bandpass filter (1–20 Hz) was applied to the signals. The single-beat (SB) method described in Lemay et al. (2007), in which QRS and T-waves are processed separately, was used to extract atrial activity on all 12-lead ECG recordings. Following application of the SB method, signals were downsampled to 50 Hz, since the frequency content of interest in atrial signals is generally below 10 Hz (Holm et al., 1998). Once downsampled signals devoid of ventricular activity were obtained, time and frequency domain ECG parameters quantifying organization and evolution of AF were calculated as described in the next section.

### 2.4. Calculation of ECG Parameters

As this study aimed to examine the temporal evolution of AF organization indices in a way that would be clinically applicable, several different factors were considered when choosing appropriate analysis methods, including the non-stationary and multi-variate nature of ECG signals recorded in AF, as well as the feasibility of a real-time implementation. Various time-frequency approaches exist for estimating frequency in non-stationary data. The cross Wigner-Ville distribution was for example used in Stridh et al. (2001) to estimate the IF on the single ECG leads *V*_1_, *V*_2_, and *V*_3_. However, these methods can only estimate frequency instantaneously insofar as their time-frequency resolution compromise allows. Additionally, these methods are not designed for multivariate applications, and so do not inherently take advantage of the redundancy that exists between signals recorded on each of the six precordial ECG leads, which can be exploited to provide better overall performance and robustness (Prudat and Vesin, 2009). Empirical mode decomposition for time-frequency analysis has been extended for multi-variate applications, and has found data-adaptive application in tracking variations in the characteristic 10-Hz μ rhythm found in EEG signals (Mandic et al., 2013). However, this method requires that signals be available in their entirety and so would not be suitable for a real-time application.

Adaptive frequency tracking algorithms consisting of both a time-varying bandpass or notch filter and an adaptive mechanism to update the filter center frequency were therefore preferred for this study, which aimed to analyze methods that could be useful in a clinical context. The delay ensued by the bandpass or notch filtering operation can be reduced through the use of a suitable infinite impulse response filter. To our knowledge, this is the first study to use a multi-lead method on ECG signals to track changes in AF organization throughout ablation. For this reason, rather than use time-frequency methods, we have chosen to use an adaptive multi-lead frequency tracking algorithm developed previously in this group. We show first the application of this algorithm for single-lead ECG signals, and then demonstrate the advantage of using the multi-lead algorithm. We have shown that adaptive, time-variant algorithms outperform methods based on time-invariant band-pass filters (BPF), which could be less accurate for extracting the signal frequency components relevant to AF (Buttu et al., 2016). Here, we used an adaptive frequency tracking scheme to extract: (i) the common oscillatory component present in multi-lead ECG signals, and (ii) the oscillations of the fundamental frequency and its first harmonic from single-lead ECG signals.

#### 2.4.1. Adaptive Frequency Tracking

The adaptive algorithm used here has been extensively described in references (Prudat and Vesin, 2009; Van Zaen et al., 2010; Buttu et al., 2013). The following is a brief description of its applicability for single, multi-signal, and harmonic frequency tracking.

The single frequency tracking algorithm has two parts, a time-varying BPF that extracts the oscillatory component present in the input signal, and an adaptive mechanism that controls the central frequency of the time-varying BPF, which is then taken as the IF. The transfer function of the time-varying BPF is defined as:

$$\begin{array}{llll} & H\left(z,n\right)=\frac{1-\beta }{2}\frac{1-z^{-2}}{1-\alpha \left(n\right)\left(1+\beta \right)z^{-1}+\beta z^{-2}}\; & \; & \; \end{array}$$

where β (0≪β < 1) controls the bandwidth of the BPF, and α(·) = *cos*(ω(·)) is the adaptive update of the central frequency of the filter. For a single input signal *x*(*n*), the output of the BPF is:

$$\begin{array}{l} y\left(n\right)=\left(1+\beta \right)\alpha \left(n\right)y\left(n-1\right)-\beta y\left(n-2\right)\\ \;+\frac{1-\beta }{2}\left[x\left(n\right)-x\left(n-2\right)\right] \end{array}$$

The central idea is to update α(*n*) to α(*n*+1) so that *y*(*n*) obeys the discrete oscillator equation. That is, the following cost function is minimized:

$$\begin{array}{ll} J\left(n\right)=E\left\{{\left[y\left(n\right)-2\alpha \left(n+1\right)y\left(n-1\right)+y\left(n-2\right)\right]}^{2}\right\},\text{where}E\left\{\cdot \right\}\; & \;\\ \text{is the expectation operator}.\; & \; \end{array}$$

In practice, the update of the central frequency of the BPF is recursively estimated using the following equation:

$$\begin{array}{lll} \alpha \left(n+1\right)=\frac{Q\left(n\right)}{2P\left(n\right)}\text{, with } & \; & \; \end{array}$$

$$\begin{array}{lll} Q\left(n\right)=\delta Q\left(n-1\right)+\left(1-\delta \right)y\left(n-1\right)\left(y\left(n\right)+y\left(n-2\right)\right)\; & \; & \;\\ P\left(n\right)=\delta P\left(n-1\right)+\left(1-\delta \right)y^{2}\left(n-1\right)\; & \; & \; \end{array}$$

where the convergence rate can be adjusted using a forgetting factor δ (0≪δ < 1). The IF of the common oscillatory component is then computed as:

$$\begin{array}{lll} f\left(n+1\right)=\arccos\frac{\alpha \left(n+1\right)}{2\pi }\; & \; & \; \end{array}$$

To extract the common frequency of *M* signals, the same BPF is used on each signal to compute individual updates as in the single-signal case. The central frequency is then updated using a weighted sum of the update terms, which are calculated separately for each signal. The computation of the weights is based on the minimization of the variance of the linear combination of the individual updates of the central frequency (Prudat and Vesin, 2009). This amounts to the same thing as giving larger weights to those signals in which the common oscillation makes up a larger part. The weighting process yields:

$$\begin{array}{lll} \alpha \left(n+1\right)=\overset{M}{\underset{m=1}{\sum }}W_{m}\left(n\right)\frac{Q_{m}\left(n\right)}{2P_{m}\left(n\right)}\; & \; & \; \end{array}$$

with weights 0 ≤ *W*_*m*_(*n*) ≤ 1, *m* = 1, …, *M* and $\overset{M}{\underset{m=1}{\sum }}W_{m}\left(n\right)=1$.

**Figure 1:**
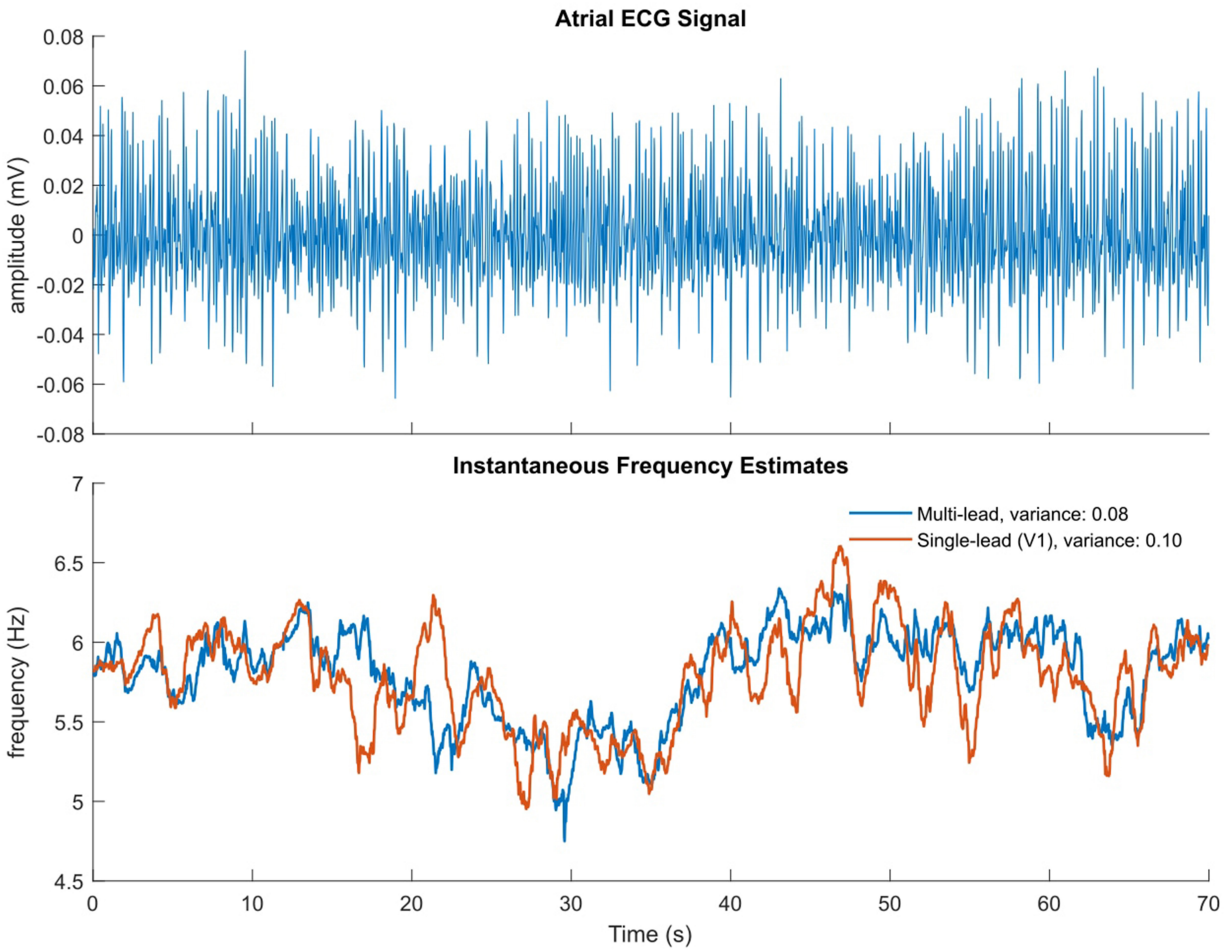
Illustrative example of multi-lead vs single-lead IF estimate. **(Top)** Atrial ECG signal with ventricular complexes removed, on which the single-lead estimate is calculated. **(Bottom)** Multi-lead IF estimate in blue, single-lead (*V*_1_) estimate in orange.

The single frequency tracker can also be extended to estimate the instantaneous fundamental frequency and extract the harmonic components in a single signal. The extension uses a separate time-varying BPF for each of the fundamental and harmonic components. Therefore, an estimate of the fundamental frequency is computed for each extracted oscillation with an adaptive mechanism. As in the multi-signal extension, a weighting procedure is applied in order to compute a global estimate of the fundamental frequency (Van Zaen et al., 2010; Buttu et al., 2013). The variances of the IF estimates obtained using the single frequency tracker and the multi-signal frequency tracker were compared for the signals used in this study, using ECG lead *V*_1_ for the single-frequency estimate, and ECG leads *V*_1_-*V*_6*b*_ as input for the multi-signal estimate. It was found that the IF estimate variance was significantly less in the multi-lead case than single-lead only. An example illustrating the reduced variance obtained with the multi-lead IF estimate compared to that of the single-lead estimate is shown in Figure 1. The multi-signal frequency tracker was therefore used in this study to obtain IF estimates.

#### 2.4.2. ECG-Based Organization Indices

Following atrial activity extraction as outlined in section 2.3, AF organization was quantified using frequency and time domain parameters. Frequency domain parameters included the IF and the adaptive organization index (AOI). The IF of the AF oscillations common to all the precordial leads (*V*_1_-*V*_6*b*_) was extracted using the multi-signal frequency tracking extension outlined above. The AOI was defined as the ratio between the power of the extracted fundamental and harmonic frequency components and total power of the input signal. The AOI is thus bounded between zero and one, with a value close to one indicating that most of the signal power is concentrated in the fundamental and first harmonic frequency components. The AOI was computed on ECG leads *V*_1_-*V*_6*b*_. The algorithm bandwidth β and update δ parameters were both set to 0.95, giving a convergence time of less than one second at a sampling frequency of 50 Hz. Time domain parameters were found separately for the extracted atrial signals on ECG leads *V*_1_-*V*_6*b*_, and included sample entropy (SampEn) (Alcaraz et al., 2010) and f-wave amplitude (FWA) (Meo et al., 2012). SampEn was calculated on the extracted atrial signals, using *m* = 2 samples within a tolerance of *r* = 0.2 applied to the signal standard deviation. The FWA was found by halving the difference of the upper and lower envelopes of the atrial signals obtained using local maxima and minima.

The IF and AOI signals were then divided into 10-s epochs, and one mean value was recorded for each epoch. For the time-domain measures, the extracted atrial signals were divided into 10-s epochs, and one SampEn and one mean FWA value was recorded for each epoch. Therefore, one mean multi-lead IF estimate and six mean AOI, six SampEn, and six mean FWA values (one for each lead) were recorded for each 10-s signal epoch, for each patient. Finally, each epoch was assigned one of the following three labels based on its temporal placement: (i) **pre-PVI**, if the epoch signal was recorded at baseline, prior to the start of the ablation, (ii) **dur-PVI** if the epoch signal was recorded during the PVI step of the procedure, (iii) **post-PVI** if the epoch signal was recorded during the CFAE or linear ablation steps.

### 2.5. Patient Group Comparison at Each Ablation Step

High DF and low OI values, as measured from the surface ECG, have been shown to predict likelihood of arrhythmia recurrence following CA (Szilágyi et al., 2018), and predictive performance has been shown to increase via the inclusion of adaptive frequency tracking organization parameters (Buttu et al., 2016). Based on these studies, here we compared the values of the IF, AOI, SampEn, and FWA at the three procedure steps outlined in section 2.3, for the three patient groups outlined in section 2.1. We calculated these parameter values on all epochs, and then averaged all available epochs for each patient, to calculate one mean value for each patient at each procedure step.

### 2.6. IF and AOI Temporal Evolution During Step-CA

**Figure 2:**
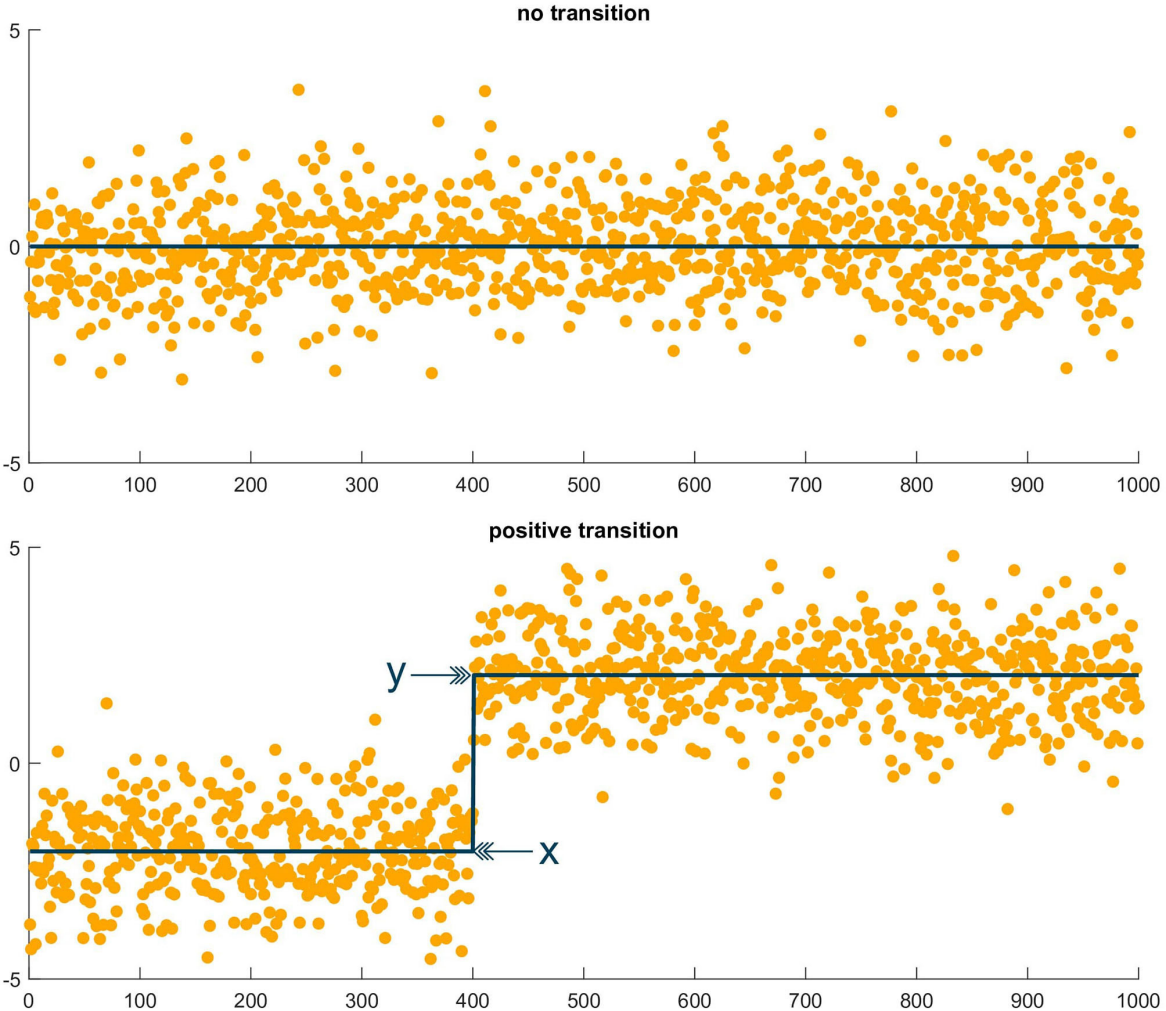
Illustrative example of MDL test. **(Top)** Sequence of random values in which the MDL test found no transition. **(Bottom)** Sequence of random values, plus step function, in which the MDL test found a transition at the step index, with *x* the mean value before the transition and *y* the mean value after the transition.

To investigate the temporal evolution of the IF and AOI during ablation, we calculated the IF (multi-lead estimate) and AOI (lead *V*_1_ only) signals for all epochs in the dur-PVI and post-PVI steps. For each patient, we then created a temporal sequence of the mean values of each of the epoch IF and AOI signals, rather than averaging epoch means together, as was done in section 2.5. Therefore, two time sequences, composed of the mean values of the IF and AOI signals found for each epoch, were available for each patient. To test for the presence of a possible transition in the mean of the sequences, we used a statistical approach based on the principle of Minimum Description Length (MDL) (Rissanen, 1978). We compared the MDL values obtained when two different models were used to model the sequence: (i) a model with two parameters (one mean and one variance), and (ii) a model with three parameters (two local means and one variance). To estimate the second model, all sample indices, except the first and last, were tested as candidates for the index of the transition, with the two means computed using respectively the samples preceding and following the candidate index. The index corresponding to the smallest residual variance was then retained as the transition index. The MDL was computed using

$$\begin{array}{lll} \text{MDL}=N\log{\hat{\sigma }}^{2}+k\log N\; & \; & \; \end{array}$$

with *N* the number of epochs, ${\hat{\sigma }}^{2}$ the variance estimate, and *k* the number of parameters, i.e., *k* = 2 for the first model, and *k* = 3 for the second model. The MDL values obtained for the first and second models were compared, and if the MDL for the first model was smaller than that of the second, no mean transition was considered to take place. If the MDL for the second model was smaller than that of the first, a significant transition was considered to be present at the retained index. We quantified the significant transitions as follows:

$$\begin{array}{lll} \text{absolute change}=y-x\; & \; & \;\\ \text{relative change}=\frac{y-x}{0.5\left(y+x\right)}\; & \; & \; \end{array}$$

where *y* was the mean value after the retained index, and *x* was the mean before the retained index. An example of the test applied to two sequences, one with a transition and one without, is shown in Figure 2. Finally, in the case that a mean change was probable, we recorded whether the change occurred in the dur-PVI or post-PVI portion of the epoch sequence.

### 2.7. Temporal Evolution of AF Organization Post-PVI

Following the analysis of the temporal evolution throughout step-CA, we took a closer look at what may happen in the post-PVI procedure step, to see whether different patterns could be found not only between the different patient groups, but also between different types of atrial arrhythmia recurrence. For each available patient, we created temporal sequences of mean epoch IF (multi-lead estimate), AOI, SampEn, and FWA (lead *V*_1_ only) values as was done for the IF and AOI in section 2.6, but here using only those epochs labeled post-PVI. We applied the statistical test described in the previous section, and placed the sequences separately into one of the following three categories, according to the type of transition reported by the test: (i) **Type 1**. AF organization increases. For IF sequences, this is indicated by negative transitions, since decreasing frequency indicates an increase in AF cycle length (AFCL), and thus a more organized arrhythmia. For SampEn, this is also indicated by negative transitions, since lower SampEn values indicate less complexity and more regularity in time. For AOI and FWA sequences, this is indicated by positive transitions. (ii) **Type 2**. AF organization decreases. For IF sequences, this is indicated by positive transitions since increasing frequency indicates shorter AFCL. For SampEn, this is also indicated by positive transitions. For AOI and FWA sequences, this is indicated by negative transitions. (iii) **Type 3**. No changes in AF organization, indicated by no transitions in the sequences. The absolute and relative changes described in the previous section were also calculated for all sequences.

### 2.8. Statistical Analysis

All numerical values are expressed as median and interquartile range (IQR). Organization index values were compared across the three groups (LTN, LTR, and NLT) for all chest leads using the Kruskal-Wallis test. Amplitudes of post-PVI relative percent changes across the three groups (LTN, LTR, and NLT) as well as by type of recurrence (AT/AFL or AF) were also compared using the Kruskal-Wallis test. Statistical comparisons for categorical variables measured using patient counts were performed using Fisher's exact test. Statistical significance was considered for *p* < 0.05.

## 3. Results

### 3.1. Study Population

A total of 40 patients were included in this study. Clinical characteristics for all patients are summarized in Table 1. The procedural endpoint was achieved in 28 patients (70%). AF was not terminated in the remaining 12 patients (NLT group), all of whom experienced an arrhythmia recurrence during follow-up. For patients in whom the procedural endpoint was reached, 8 (20%) remained in SR (LTN group) throughout the follow-up period following a single procedure. Arrhythmia recurrence was observed in the remaining 20 patients (LTR group). In the LTR and NLT groups, recurrence occurred as AF (*n* = 7) and as AT/AFL (*n* = 25) on average 7 ± 10 months after the index procedure. Note that the sustained AF duration of the NLT group (median [IQR]: 22 [14; 39] months) was significantly longer than that of the LTN and LTR groups (20 [10; 24] and 13 [12; 24] months, respectively). The cumulative ablation time was also significantly different for each group, being the longest for the NLT group (76 [61; 81] min.), followed by the LTR group (55 [50; 60] min.), and finally shortest for the LTN group (40 [29; 54] min.). The mean follow-up duration for the study population was 32 ± 14 months, and at the end of the follow-up period, 34 (85%) patients were in SR without (28/34, 82%) and with amiodarone (6/34, 18%), with a mean number of 2 ± 1 CA procedures per patient.

### 3.2. ECG Parameters Before and During Ablation

**Figure 3:**
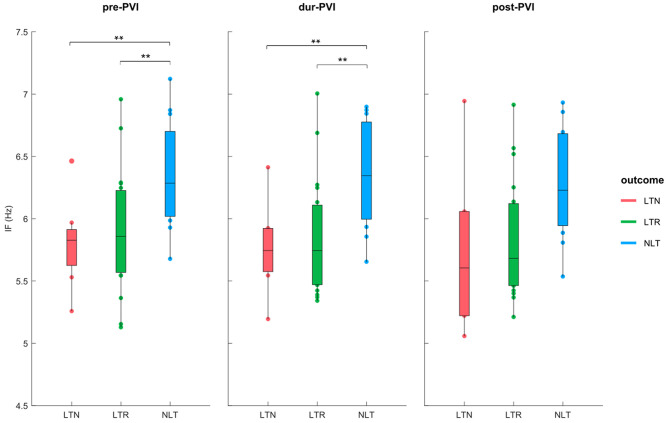
IF at pre-PVI, dur-PVI, and post-PVI for all patient groups. Significant differences between groups at each ablation step are displayed; **p < *0.01*.

**Figure 4:**
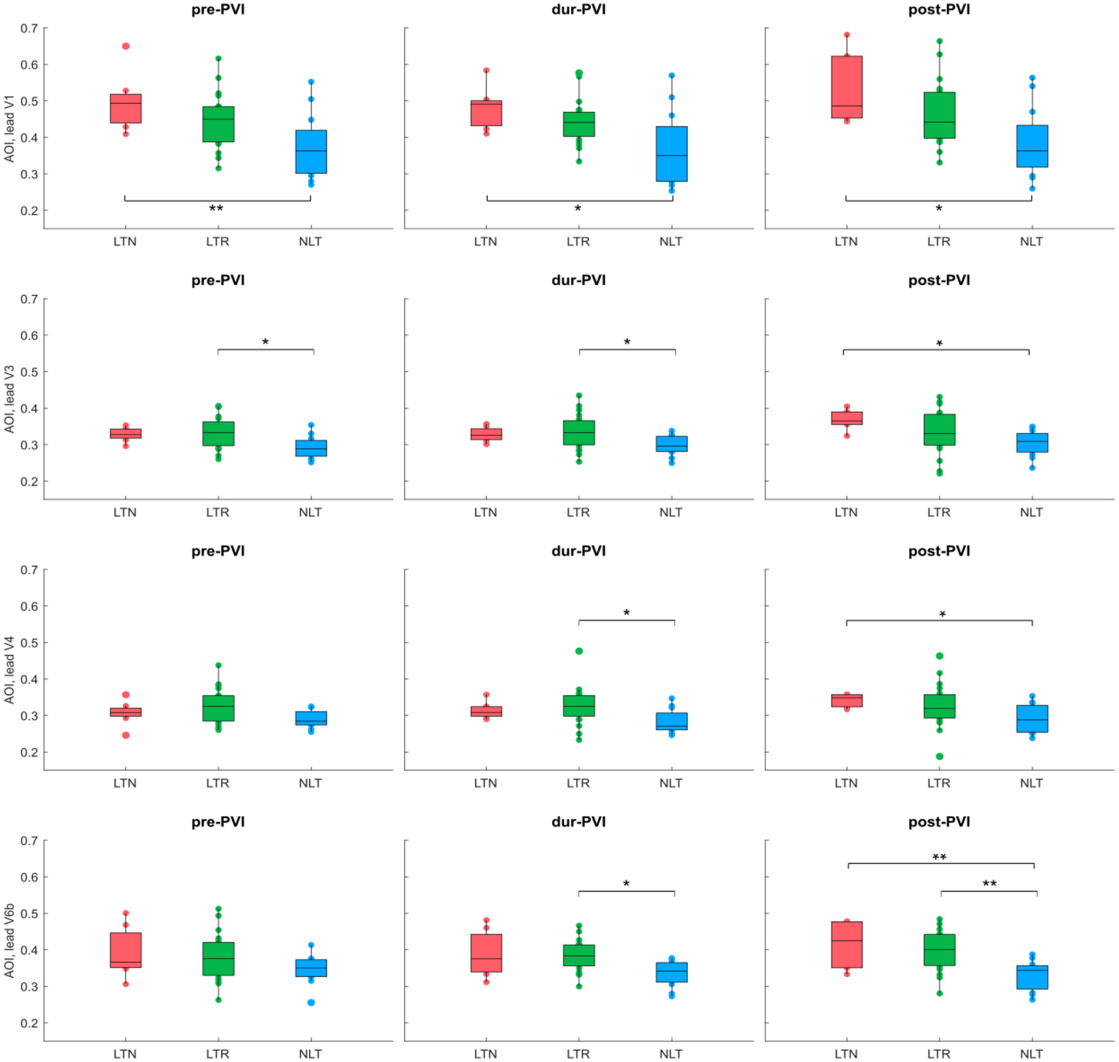
AOI calculated on leads *V*_1_, *V*_3_, *V*_4_, and *V*_6*b*_ at pre-PVI, dur-PVI, and post-PVI for all patient groups. Significant differences between groups at each ablation step are displayed; *p < *0.05*, **p < *0.01*. Leads *V*_2_ and *V*_5_ not shown, as results were not significant on these leads.

Figure 3 shows IF values for all patients at pre-PVI, dur-PVI, and post-PVI, and grouped as LTN, LTR, or NLT, to analyze both differences between patient groups and within the same patient group at different procedure steps. Figure 4 reports the same information for the AOI values calculated on leads *V*_1_, *V*_3_, *V*_4_, and *V*_6*b*_. The remaining precordial leads did not display statistical significance (data not shown). Median IF values observed in the LTN and LTR patient groups were significantly smaller (*p* < 0.01) than those observed in the NLT patient group at pre-PVI and dur-PVI. Significant differences at all steps were observed between the LTN and NLT patient groups only on lead *V*_1_. Other leads displayed varying significant differences between different combinations of groups and ablation steps. Significant differences between the LTN and NLT and the LTR and NLT groups in the same procedure step were observed only on *V*_6*b*_, post-PVI. No significant differences were observed between the IF and AOI calculated at the pre-PVI, dur-PVI, and post-PVI procedure steps within a single patient group (LTN, LTR, or NLT). Additionally, no significant differences could be observed between patient groups nor within the same group between procedure steps for the SampEn and FWA indices, for which results can be found in the Supplementary Materials. These results suggest that patients in whom CA did not successfully terminate AF displayed significantly lower levels of AF organization both prior to and throughout the CA procedure. In the following sections, the AOI, SampEn, and FWA indices will be reported for lead *V*_1_ only, as this was the lead to display significant differences between groups most consistently.

### 3.3. Temporal IF and AOI Evolution Throughout Step-CA

**Figure 5:**
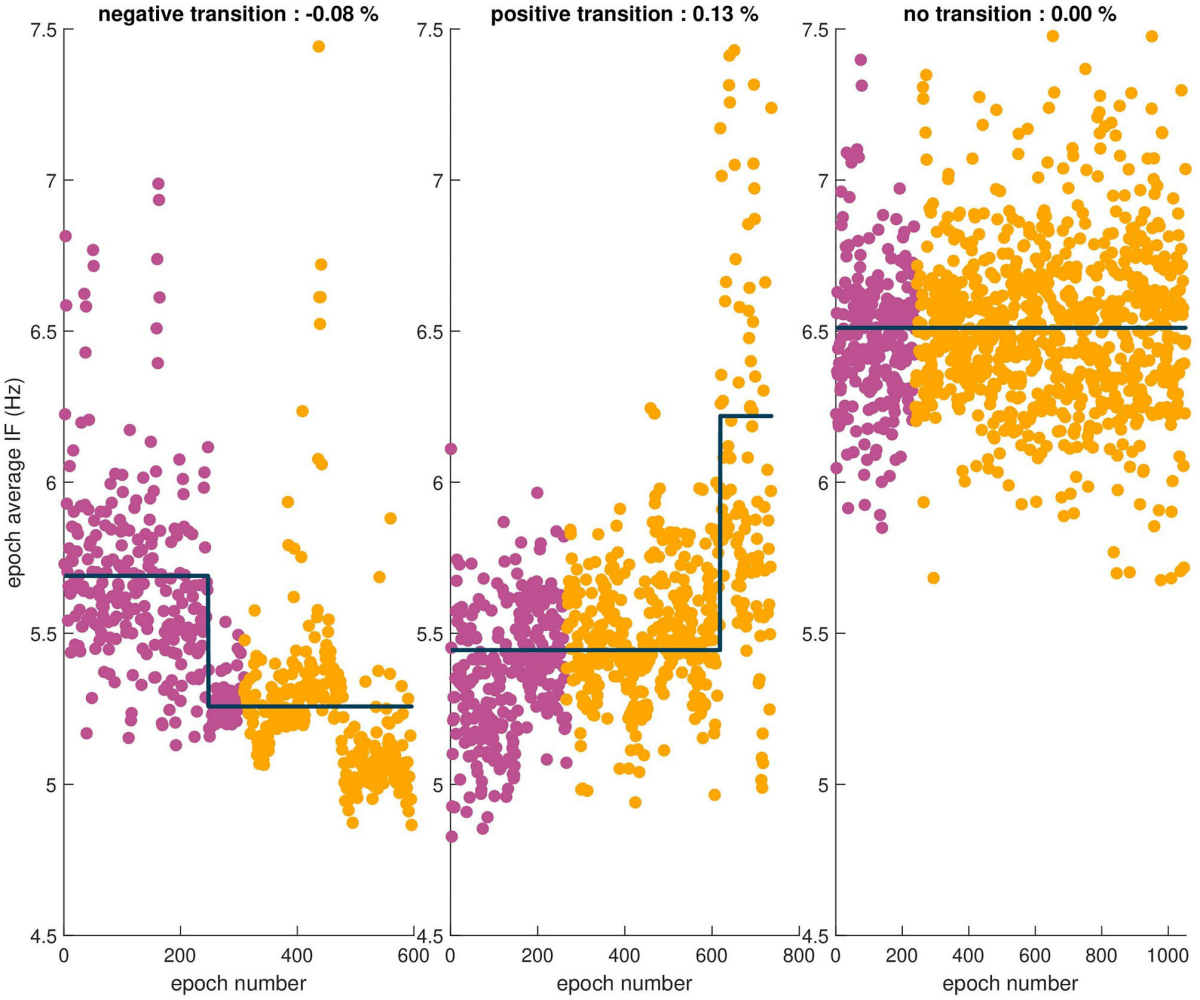
Sequences of epoch average IF values for three patients. The observed relative percent change is indicated in the title of each example. From left to right, a negative transition in the mean, a positive transition in the mean, and finally no transition observed in the mean of the sequence.

Several examples showing transitions, or lack thereof, in the means of epoch sequences reported by the MDL test are shown in Figure 5. Using the MDL statistical test, a transition in the mean of the sequence of the combined epoch IF values at dur-PVI and post-PVI was observed in 33 out of 40 patients. A transition was observed in 7/8 LTN patients, of which 2 dur-PVI and 5 post-PVI. A transition was observed in 16/20 LTR patients, of which 6 dur-PVI and 10 post-PVI. Finally, a transition was observed in 10/12 NLT patients, of which only 1 dur-PVI and 9 post-PVI. These results suggest that step-CA led to changes in AF dynamics in most patients; however, no significant association was found between the timing of the change (dur-PVI or post-PVI) and the different patient groups.

### 3.4. Temporal Evolution of AF Organization Post-PVI

**Figure 6:**
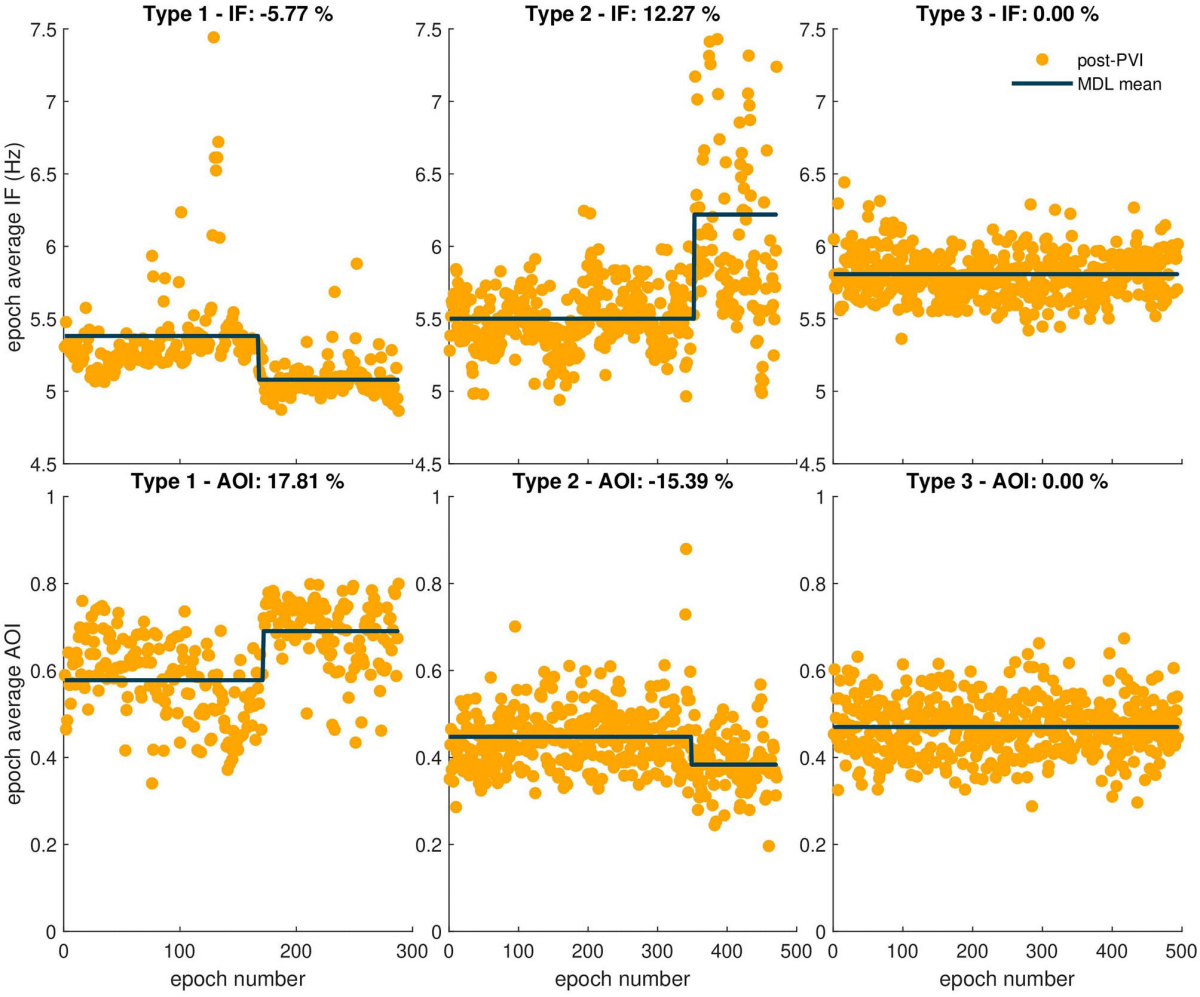
Three types of AF organization. The observed relative percent change is indicated in the title of each example. The first column shows examples of Type 1, increasing AF organization, indicated by a negative IF transition in the first row and a positive AOI transition in the second row. The second column shows examples of Type 2, decreasing AF organization, indicated by a positive transition in the IF epoch sequence, and negative transition in the AOI epoch sequence. Finally, the third column shows examples of Type 3, no change in AF organization, indicated by no transitions in either sequence.

**Figure 7:**
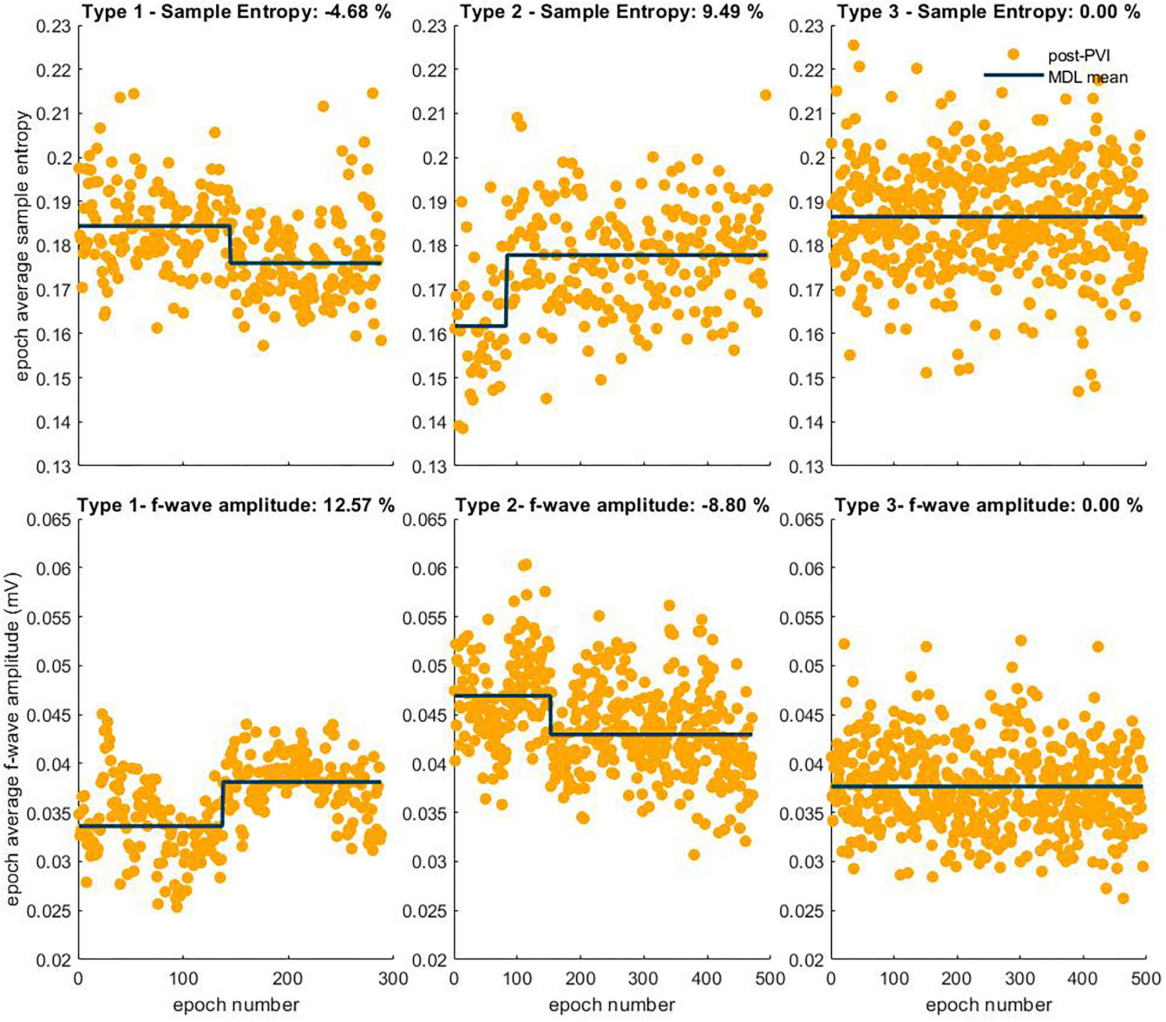
Three types of AF organization. The observed relative percent change is indicated in the title of each example. The first column shows examples of Type 1, increasing AF organization, indicated by a negative SampEn transition in the first row and a positive FWA transition in the second row. The second column shows examples of Type 2, decreasing AF organization, indicated by a positive transition in the SampEn sequence, and negative transition in the FWA epoch sequence. Finally, the third column shows examples of Type 3, no change in AF organization, indicated by no transitions in either sequence.

Atrial fibrillatory waves were not consistently observed in the post-PVI ECG signals of three patients. Since this precluded reliable atrial activity extraction for a sufficient number of epochs, the data from these patients were excluded from this part of the analysis. Additionally, two LTN patients reached the procedural endpoint dur-PVI; therefore, post-PVI analysis was not feasible. Figures 6, 7 show examples of the three types of AF organization outlined in section 2.7 for IF, AOI, SampEn, and FWA (Type 1: increasing organization; Type 2: decreasing organization; Type 3: no change in organization).

Tables 2, 3 show relative percent changes in respectively IF and AOI, and SampEn and FWA, post-PVI, according to ablation outcome. Ablation within the LA (CFAE and linear ablation) led to increases in AF organization in most patients [Type 1: decrease in IF, 24/35 (68%); increase in AOI, 25/35 (71%); decrease in SampEn, 21/35 (60%)]. Increases in FWA were observed in 16/35 (46%) of patients. However, no association between Type 1 transitions and procedural ablation outcomes was found. No significant differences were observed in the relative percent changes in the IF, AOI, SampEn, and FWA epoch sequences between the LTN, LTR, and NLT patient groups.

**Table 2:** Relative changes in IF and AOI post-PVI, according to ablation outcomes, expressed as median with [25th; 75th] percentiles.

	**LTN**		**LTR**		**NLT**	
	***n*** **=** **6**		**n** **=** **18**		**n** **=** **11**	
	**IF**	**AOI**	**IF**	**AOI**	**IF**	**AOI**
Rel. change %	−2 [−4; 16]	15 [11; 18]	−6 [−7; 0]	14 [0; 30]	−2 [−5; −1]	10 [2; 13]
Type 1	3 (50%)	5 (83%)	13 (72%)	12 (67%)	8 (73%)	8 (73%)
Type 2	2 (33%)	1 (17%)	3 (17%)	3 (17%)	1 (9.1%)	1 (9.1%)
Type 3	1 (17%)	0	2 (11%)	3 (17%)	2 (18%)	2 (18%)

*Patient counts and percentages of total count are indicated for each type of transition*.

**Table 3:** Relative changes in SampEn and FWA post-PVI, according to ablation outcomes, expressed as median with [25th; 75th] percentiles.

	**LTN**		**LTR**		**NLT**	
	***n*** **=** **6**		***n*** **=** **18**		***n*** **=** **11**	
	**SampEn**	**FWA**	**SampEn**	**FWA**	**SampEn**	**FWA**
Rel. change %	−3.8 [−4.7; 0]	−0.21 [−16; 13]	−4.3 [−7.5; 0]	0 [−2.5; 14]	−2.1 [−3; 0]	3.7 [0; 5.7]
Type 1	4 (67%)	3 (50%)	11 (61%)	7 (39%)	6 (55%)	6 (55%)
Type 2	1 (17%)	3 (50%)	0	5 (28%)	0 (0%)	2 (18%)
Type 3	1 (17%)	0	7 (39%)	6 (33%)	5 (45%)	3 (27%)

*Patient counts and percentages of total count are indicated for each type of transition*.

Table 4 shows the distribution of the three categories of temporal evolution in the IF and AOI sequences, according to the form of recurrence observed during the follow-up period after a single ablation procedure. Increases in AF organization during ablation, as indicated by a decrease in IF and/or increase in AOI, were more frequent in patients whose arrhythmia recurred as AT/AFL than in patients whose arrhythmia recurred as AF [AT/AFL vs. AF, Type 1: decrease in IF, 19/23 (83%) vs. 2/6 (33%); increase in AOI, 18/23 (78%) vs. 2/6 (33%); *p* < 0.05]. Median relative percent changes in IF and AOI showed significantly greater amplitudes in AT/AFL recurrences than in AF recurrences (IF: −5 [−7; −2]% vs. 0 [−2; 0]%; AOI: 13 [5; 29]% vs. 0 [−6; 8]%; *p* < 0.05). This suggests that if AF reorganization occurred post-PVI, then recurrence occurred as an organized arrhythmia, such as AFL or AT. However, little or no post-PVI reorganization may have led to recurrence as a disorganized arrhythmia, i.e., AF. The same information for the SampEn and FWA variables, for which no statistically significant differences between patient groups nor between types of arrhythmia recurrences could be found, is included in the Supplementary Materials.

**Table 4:** Relative changes in IF and AOI post-PVI, according to form of recurrence, expressed as median with [25th; 75th] percentiles.

	**AT/AFL**		**AF**	
	***n*** **=** **23**		***n*** **=** **6**	
	**IF**	**AOI**	**IF**	**AOI**
Rel change %	−5 [−7; −2]^a^	13 [5; 29]^a^	0 [−2; 0]^a^	0 [−6; 8]^a^
Type 1	19 (83%)^b^	18 (78%)	2 (33%)	2 (33%)
Type 2	3 (13%)	2 (9%)	1 (17%)	2 (33%)
Type 3	1 (4%)	3 (13%)	3 (50%)	2 (33%)

a *AT/AFL vs AF (p < 0.05)*.

b *Type 1 transition associated with recurrence (p < 0.05)*.

*Patient counts and percentages of total count are indicated for each type of transition*.

## 4. Discussion

This exploratory study contains several findings, including: (i) patients in whom ablation failed to terminate AF and to restore long-term SR displayed the lowest AF organization level as indicated by the highest IF and lowest AOI values, (ii) IF and AOI mean value transitions were more likely to occur post-PVI than dur-PVI, and (iii) AF reorganization during post-PVI was associated with organized arrhythmia recurrence as AT/AFL, while lack of AF reorganization was associated with disorganized arrhythmia recurrence as AF.

It has been suggested previously that high baseline DF and low OI values measured from the surface ECG are likely to predict arrhythmia recurrence after CA (Okumura et al., 2012; Szilágyi et al., 2018; Murase et al., 2020). In this study, an analysis of the median trends at each of the pre-PVI, dur-PVI, and post-PVI ablation steps confirmed higher IF and lower AOI values in NLT patients at pre-PVI, for whom the step-CA procedure failed to terminate pers-AF. This analysis also revealed that these higher IF and lower AOI values persisted at the dur-PVI and post-PVI steps. However, we found that for any individual patient group (LTN, LTR, or NLT), the median IF and AOI values did not change significantly between the pre-PVI, dur-PVI, and post-PVI steps.

The temporal evolution of surface ECG and intracardiac EGM measures throughout CA has been previously studied. In Forclaz et al. (2011), AFCL was observed to increase between progressive steps of post-PVI ablation in pers-AF patients, regardless of whether the step-CA procedure successfully terminated their AF. However, an increase in a temporal regularity index similar to the OI was found only in patients with termination. In addition, patients with the largest increases in the temporal regularity index more commonly experienced long-term freedom from arrhythmia during a follow-up period. A progressive increase in AFCL was observed at subsequent ablation steps in Calò et al. (2006), and a gradual increase in AFCL between ablation steps was associated with AF termination by ablation in D O'Neill et al. (2006). Lankveld et al. (2016) investigated temporal changes in the values of DF estimated from 10-s ECG recordings obtained after each step performed in step-CA, for patients with pers-AF. An overall decrease in DF was found after each ablation step, with the highest values observed pre-ablation, decreasing following PVI, and further decreasing following LA ablation. The exact changes in the DF depended on whether left and/or right-sided ablation were performed. These findings are suggestive of a progressive increase in AF organization throughout CA; however they do not reveal, at the level of surface ECG, which CA steps may contribute most to this increase. Our study investigated this by extending the analysis to include many ECG epochs for each of the pre-PVI, dur-PVI, and post-PVI ablation steps, rather than only one segment after each procedure step as in Lankveld et al. (2016). In the present study, when the temporal evolution of AF complexity parameters dur-PVI and post-PVI was estimated statistically using a test for mean value transition based on the principle of MDL, IF, and AOI mean transitions were observed in a large majority of patient epoch sequences. It was also observed that most of these transitions occurred in the post-PVI step. When mean value transitions were estimated on sequences composed of only post-PVI epoch means, most of them could be categorized as increasing AF organization, i.e., negative transitions in the IF sequences, indicative of a lengthening in the AFCL, and positive transitions in the AOI sequences. This suggests that it was CFAE and linear ablation, rather than PVI, that was associated with increasing AF organization, and therefore that AF substrate was more likely located in the LA, and not the pulmonary veins. The study performed in Ha¨ıssaguerre et al. (2005) found that the largest increments in the gradual prolongation of the AFCL were observed after ablation of the PV-LA junction, inferior LA/CS interface, and left atrial appendage (LAA). Our study suggests that this effect may be observed at the surface ECG level. The differences in the amplitudes of the transitions were not significantly linked to the LTN, LTR, and NLT patient groups. However, it was found that increases in AF organization post-PVI occurred more often in LTR and NLT patients who had a recurrence of AT or AFL, both organized arrhythmias. In contrast, when no AF reorganization was observed post-PVI, disorganized arrhythmia recurrence as AF was more common. These findings suggest that ECG-based AF organization indices could help to assess the risk of later recurrence in the form of AT/AFL or AF after a single step-CA procedure.

The potential applications of analyzing the temporal evolution of ECG parameters throughout CA are manifold, including further insight into the interaction of CA with local substrate and the efficacy of an ongoing ablation.

## 5. Study Limitations

The small population size in this study may limit its predictive power. In addition, because this was a single-center study in which a single operator (EP) performed all ablation procedures, results may not extend to other centers. Importantly however, the study included consecutive patients undergoing radio-frequency ablation procedures for treatment of pers-AF; therefore, no selection bias was introduced. Also limiting our study was the recording of ECG signals only while patients were under general anesthesia (GA), and this may have affected atrial activity dynamics. However, patient response to step-CA was assessed using relative mean transitions in temporal ECG-based organization indices throughout ablation, reducing bias that could have been introduced by varying patient responses to GA. Importantly, our study was intended to identify (before and during ablation) ECG-based markers to characterize the relationship of the AF substrate to procedural and clinical outcomes. Our study is however missing a comparative analysis of suitable measures designed for intracardiac electrograms that would strengthen our results. A final limitation was the definition of the clinical endpoint as a successful ablation after a single procedure, which might minimize the success rate of step-CA after multiple procedures (Scherr et al., 2015). Since most recurrences occurred as AT/AFL, using a single-procedure ablation success as a clinical endpoint was aimed at lowering the bias due to repeat ablation procedures. The high recurrence as AT/AFL may be a consequence of the extensive ablation in the index procedure, which included PVI, defragmentation, and lines. Any remaining gaps in these lesions may favor the emergence of AT/AFL. Importantly, recurrence occurring as AT/AFL may be an expression of both the ablation extent and the level of bi-atrial remodeling (Yang et al., 2017).

## 6. Conclusion

This study suggests that non-invasive ECG measures may be used pre-PVI to show the lowest levels of AF organization in NLT patients who do not respond well to step-CA. These measures are not however suitable for predicting which patients will experience an arrhythmia recurrence following a single step-CA procedure.

Additionally, investigation of the temporal evolution of ECG-based indices during step-CA bears merit for consideration as a clinical tool to assess the progression of ongoing ablation procedures. Ablation following PVI within the LA (CFAEs and linear ablation) could be associated with increasing AF organization, particularly in those patients who may experience an arrhythmia recurrence as AT/AFL rather than AF. Patterns of temporal evolution in adaptive ECG measures could not be shown to distinguish pers-AF patients who did or did not experience an arrhythmia recurrence.

## Data Availability Statement

The data analyzed in this study is subject to the following licenses/restrictions: Data were collected in collaboration with industry. Requests to access these datasets should be directed to anna.mccann@epfl.ch.

## Ethics Statement

The studies involving human participants were reviewed and approved by the Lausanne University Hospital Human Research Ethics Committee. The patients/participants provided their written informed consent to participate in this study.

## Author Contributions

AM, J-MV, EP, and AL conceived and designed the study and were in charge of manuscript writing. AM, J-MV, and AL performed the analyses. EP provided a clinical interpretation of the results. EP, CS, and LR contributed to data acquisition. All authors contributed to the article and approved the submitted version.

## Conflict of Interest

The authors declare that the research was conducted in the absence of any commercial or financial relationships that could be construed as a potential conflict of interest.
