# Supplementary material for: ECG-Based Indices to Characterize Persistent Atrial Fibrillation Before and During Stepwise Catheter Ablation
Source: Front Physiol. 2021 Mar 30;12:654053. doi: 10.3389/fphys.2021.654053 (PMC8042333; doi:10.3389/fphys.2021.654053)
Supplement: Supplementary file 1 [file Table_1.docx]

**Supplementary Results**

**Mean SampEn and FWA before and during ablation**

Supplementary Figures 1 and 2 show respectively sample entropy (SampEn) and f-wave amplitude (FWA) values calculated on ECG leads V1-V6b for all patients at pre-PVI, dur-PVI, and post-PVI, and grouped as LTN, LTR, or NLT, to analyze differences between patient groups and within the same patient group at different procedure steps. No significant differences were observed for these variables.

**Temporal SampEn and FWA post-PVI**

Supplementary Table 1 shows the distribution of the three categories of temporal evolution in the SampEn and FWA sequences, according to the form of recurrence observed during the follow-up period after a single ablation procedure. No significant associations between changes in AF organization and type of recurrence were found for these variables.


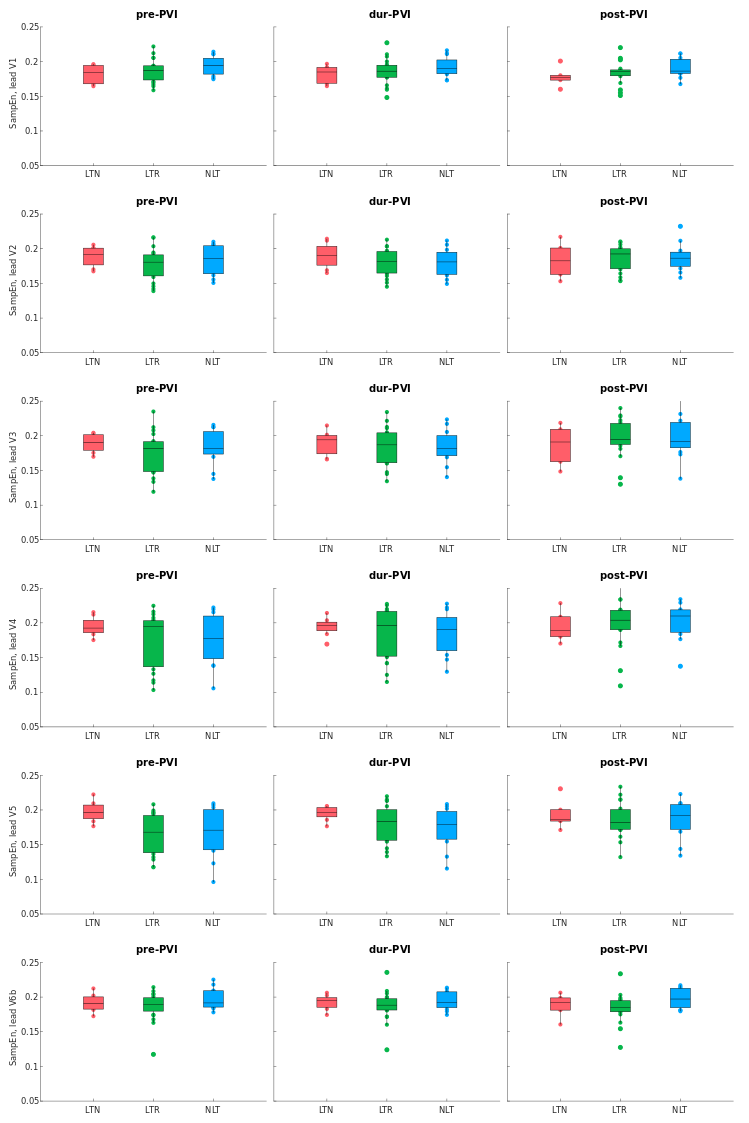


Supplementary figure 1: SampEn calculated on leads V1-V6_b_ at pre-PVI, dur-PVI, and post-PVI for all patient groups. No significant differences were found between groups at the same procedure step, nor between the same group at different procedure steps.


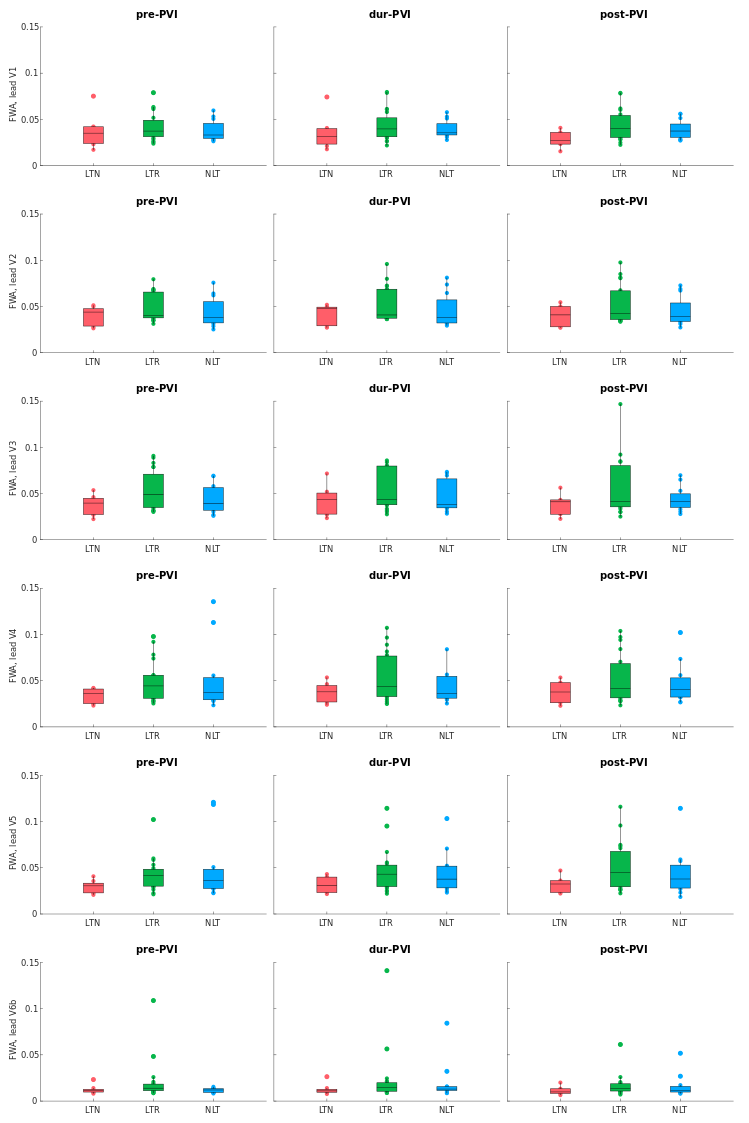


Supplementary figure 2: f-wave amplitude (FWA) expressed in mV, calculated on leads V1-V6_b_ at pre-PVI, dur-PVI, and post-PVI for all patient groups. No significant differences were found between groups at the same procedure step, nor between the same group at different procedure steps.

Supplementary Table 1. Relative changes in SampEn and FWA post-PVI, according to form of recurrence, expressed as median with [25^th^; 75^th^] percentiles. Patient counts and percentages of total count are indicated for each type of transition. No significant differences were found for either variable between AT/AFL and AF.

|  | AT/AFL  n = 23 | | AF  n = 6 | |
| --- | --- | --- | --- | --- |
|  | SampEn | FWA | SampEn | FWA |
| Rel. change % | -2.9 [-5.8; 0] | 0 [-6.6; 15] | -2.2 [-3; 0] | 0 [0; 5.8] |
| Type #1 | 14 (61%) | 11 (48%) | 3 (50%) | 2 (33%) |
| Type #2 | 0 | 6 (26%) | 0 | 1 (17%) |
| Type #3 | 9 (39%) | 6 (26%) | 3 (50%) | 3 (50%) |
